# A Nationwide Study of the Effects of Parental Income and Education on Infant Health at Birth in The Netherlands: Mediating Role of Pollution and Pesticides

**DOI:** 10.23889/ijpds.v11i4.3809

**Published:** 2026-07-06

**Authors:** Nadya Ali, Javier Garcia Bernardo

**Affiliations:** 1 Tilburg University, Tilburg, Netherlands; 2 Utrecht University, Utrecht, Netherlands

## Background

Socioeconomic gradients in birth outcomes are well documented, yet most studies classify households as above or below a fixed poverty threshold, potentially overlooking adverse birth outcomes among infants born into families just above that line. We examined non-linear associations between household income and infant health at birth across the Dutch population, and the moderating role of maternal education. Additionally, environmental exposure to pesticides and air pollution was investigated as a possible mediator of that effect.

## Methods

In this pre-registered, nationwide study, we assessed gestational age (GA), birth weight, small-for-gestational-age (SGA) birth risk, and neonatal mortality among  singleton pregnancies in the entire Dutch population between 2012 and 2020 (n = 636,680). Generalized additive models estimated non-linear income–outcome associations, stratified by maternal education. Formal mediation analyses tested whether pollution (NO_2_; PM_2.5_), and residential exposure to pesticides mediated the income–birth outcome association. Models were adjusted for maternal age, GA (where applicable), urbanity, ethnicity, parity, and infant sex.

## Results

Higher income was associated with longer GA, higher birth weight, lower SGA and neonatal mortality. Rather than plateauing at the poverty threshold, the effect did not level off until income levels well above the median. High maternal education largely buffered the income effect for GA, birth weight, and SGA, but not for neonatal mortality.

## Interpretation

Based on the findings from this study, the official poverty threshold does not correspond to the income level at which infant health disparities become negligible.

